# Supplementary material for: Energetic robustness to large scale structural fluctuations in a photosynthetic supercomplex
Source: Nat Commun. 2023 Aug 2;14:4650. doi: 10.1038/s41467-023-40146-8 (PMC10397321; doi:10.1038/s41467-023-40146-8)
Supplement: Supplementary file 1 — Supplementary Information [file 41467_2023_40146_MOESM1_ESM.pdf]

Supplementary information for: Energetic robustness to large scale structural fluctuations in a photosynthetic supercomplex

Dvir Harris[1,†], Hila Toporik[2,3,4†], Gabriela S. Schlau-Cohen[1,*], Yuval Mazor[2,3,*]

[*]Corresponding authors - gssc@mit.edu, ymazor@asu.edu

[†]These authors contributed equally to this work

[1]Department of Chemistry, Massachusetts Institute of Technology,
77 Massachusetts Avenue, Cambridge, Massachusetts 02139, USA

[2]Biodesign Institute, School of Molecular Sciences, Arizona State University, Tempe, AZ 85801, USA

[3]School of Molecular Sciences, Arizona State University, Tempe, AZ, USA

[4]Faculty of Agriculture, Food and Environment, The Hebrew University of Jerusalem, Rehovot, Israel

Contents

I. Supplementary Figures 3

II. Supplementary Tables 12

Supplementary Figures

1. Image quality and processing strategy. 3
2. Particle views and map examples. 4
3. Mask optimization. 5
4. Hexamer mask placement optimization. 6
5. Comparing principal components across the complete PSI-IsiA. 7
6. combining principle components to whole complexes. 8
7. Ensemble characterization of IsiA-PSI. 9
8. Additional transient single particles intensity traces. 9
9. Lifetime and amplitude distributions of slow component in biexponential states. . 10
10. IsiA Chls at the PSI-IsiA interface. 10
11. Sum of rates at the PSI-IsiA interface. 11
12. Specific chlorophylls contribution to energy transfer rate 11

Supplementary Tables

1. Cryo-EM data collection, refinement and validation statistics 12
2. Fastest IsiA-PSI FRET pairs in selected conformations of PC1 13

I. Supplementary Figures

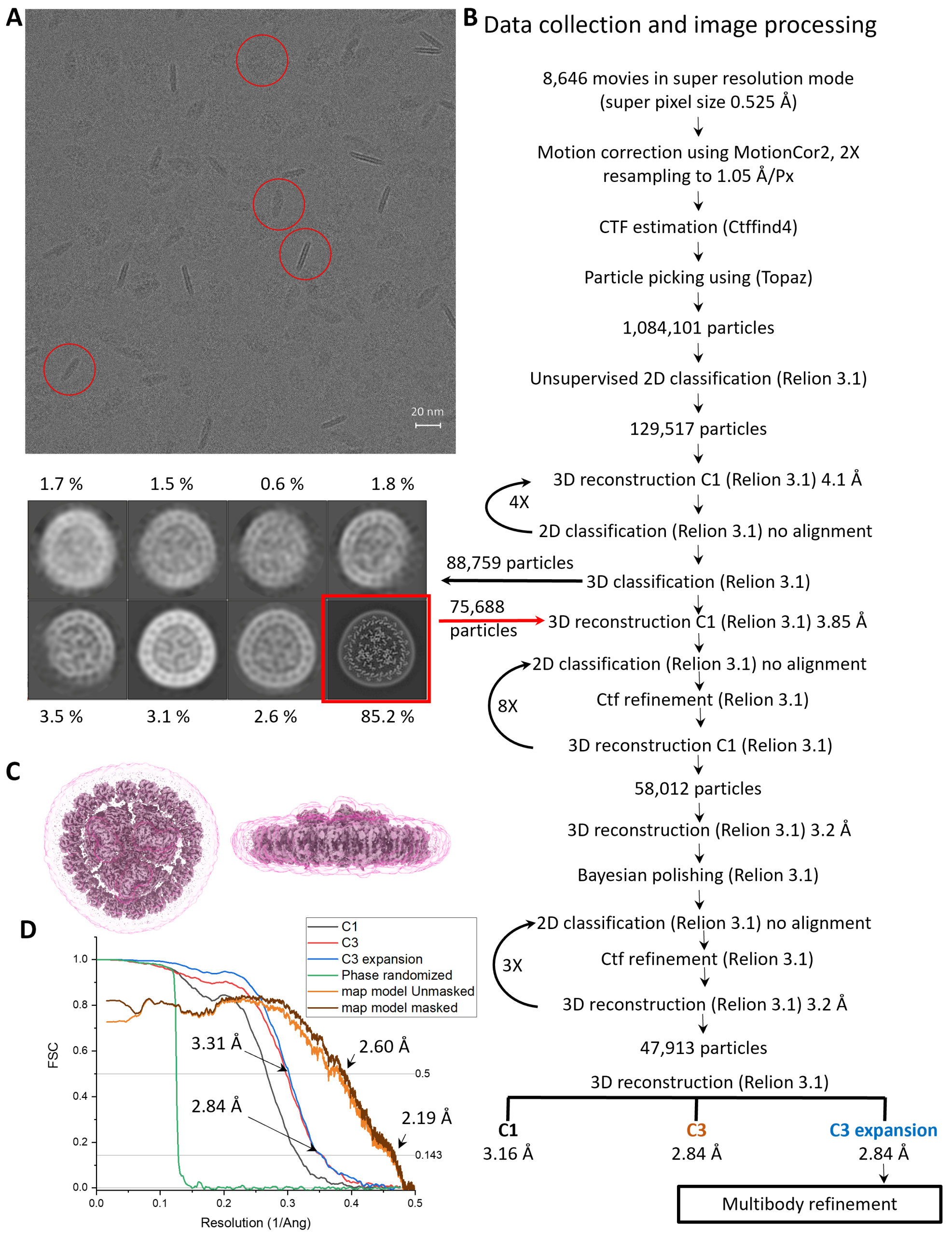

Supplementary Figure 1: Image quality and processing strategy. **(A)** A representative micrograph (1 of 8,646). PSI-IsiA particles are visible in different orientations as projections in vitreous ice on the grid. **(B)** Data collection and image processing strategy before Multibody refinement, further details can be found in the material and method section. The final particle dataset highlighted in the red square. **(C)** Mask used in the during data processing (light pink) and the final reconstructed particle (grey). **(D)** FSC curves for relion refinement runs employing C1, C3 symmetry and C3 expanded data set. In orange and dark brown, FSC between the final model and map calculated by phenix.

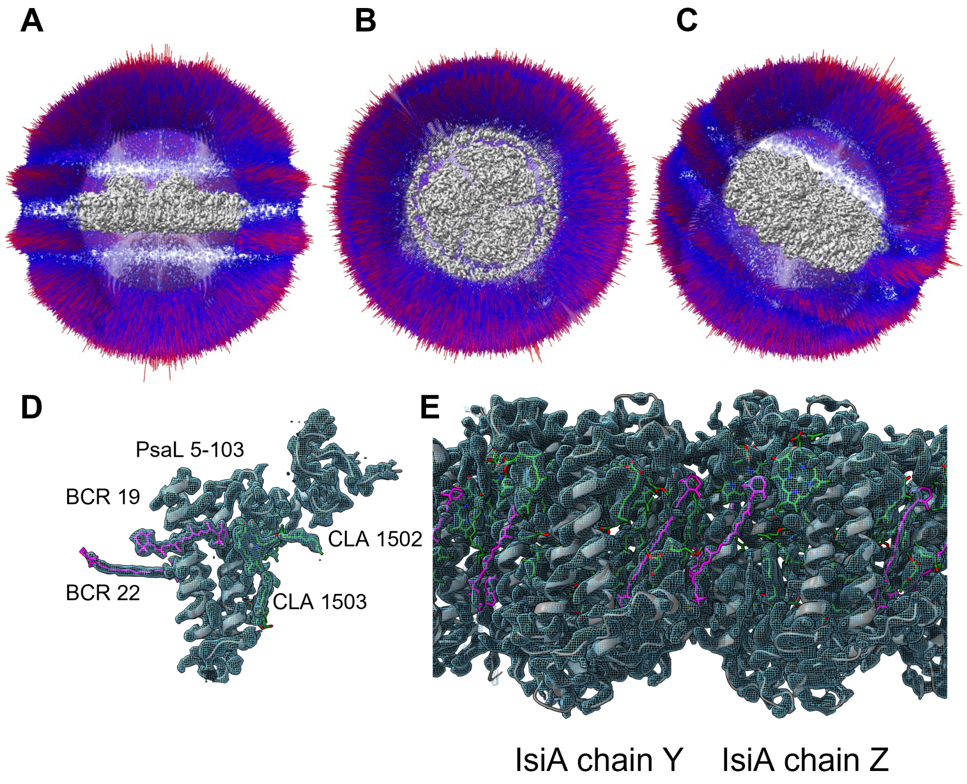

Supplementary Figure 2: Particle views and map examples. Distribution of views in the final refinement run caried out on a C3 expanded data set. The image was slice to remove some of the views to allow visualization of the PSI-IsiA particle. **(A)** View from the membrane plane. **(B)** View from the top of the membrane plane. **(C)** View oriented 45° to the membrane plane. **(D)** Map around the PSI subunit PsaL, showing amino acids 5-105, chlorophylls 1502 and 1503 and two β-carotenes. **(E)** Two IsiA monomers viewed from the membrane plane (chains Y and Z, IsiA positions 'b' and 'c').

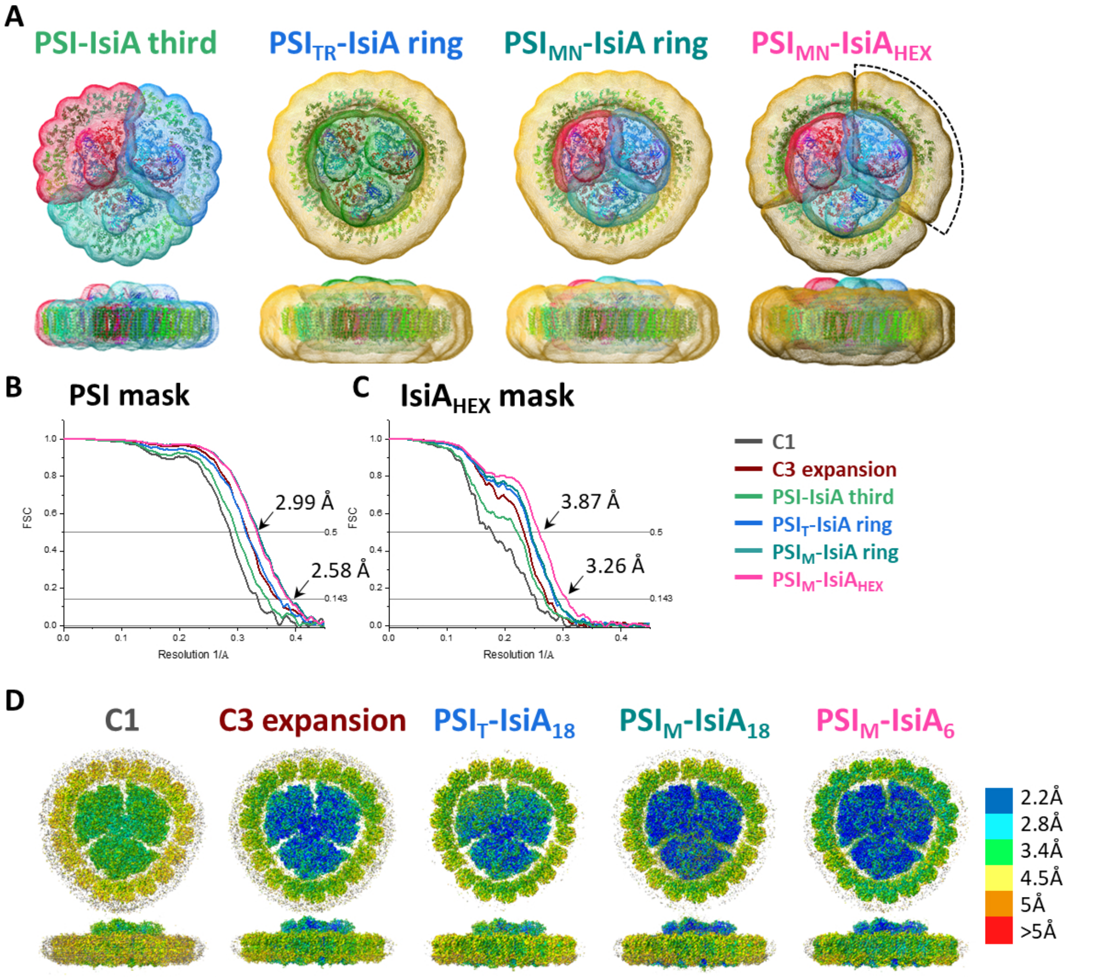

0.01	0.01	0.009	0.009	0.009	PSI map Level
0.008	0.008	0.007	0.007	0.007	IsiA map Level

Supplementary Figure 3: Mask optimization. (A) four different masking options were examined, all presented as semi-transparent surfaces viewed from the stromal side with the PSI-IsiA model enclosed. Four options were tested (left to right): PSI-IsiA thirds, PSI trimer and IsiA ring, PSI monomers and IsiA ring, PSI monomers and IsiA hexamers. The black dotted line (right most part) marks the masks used to calculate the FSC in B and C. **(B)** FSC curves on a PSI monomer using the different masks. **(C)** FSC curves on a IsiA hexamer using the different masks. **(D)** Local resolution maps of all reconstructions.

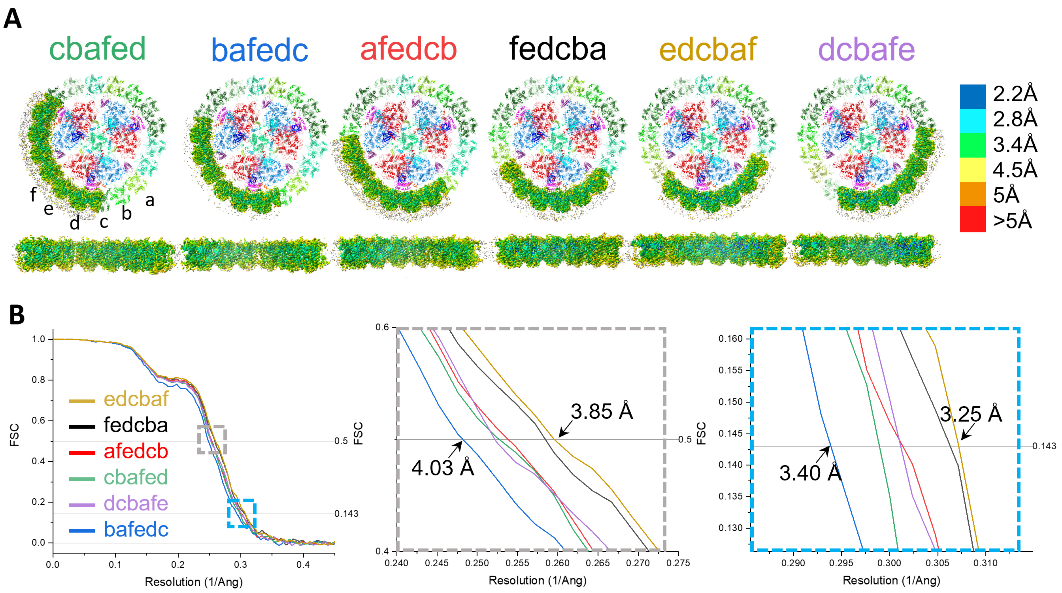

Supplementary Figure 4: Hexamer mask placement optimization. **(A)** Local resolution maps around the six tested mask positions. Top - viewed from the luminal side, bottom – a side view of each IsiA hexamer viewing from the PSI timer postion "outwards". The order of the IsiA monomers is indicated above. **(B)** FSC curves on an IsiA hexamers using the different masks. Curves around the FSC 0.5 and 0.143 are magnified in grey and blue squares.

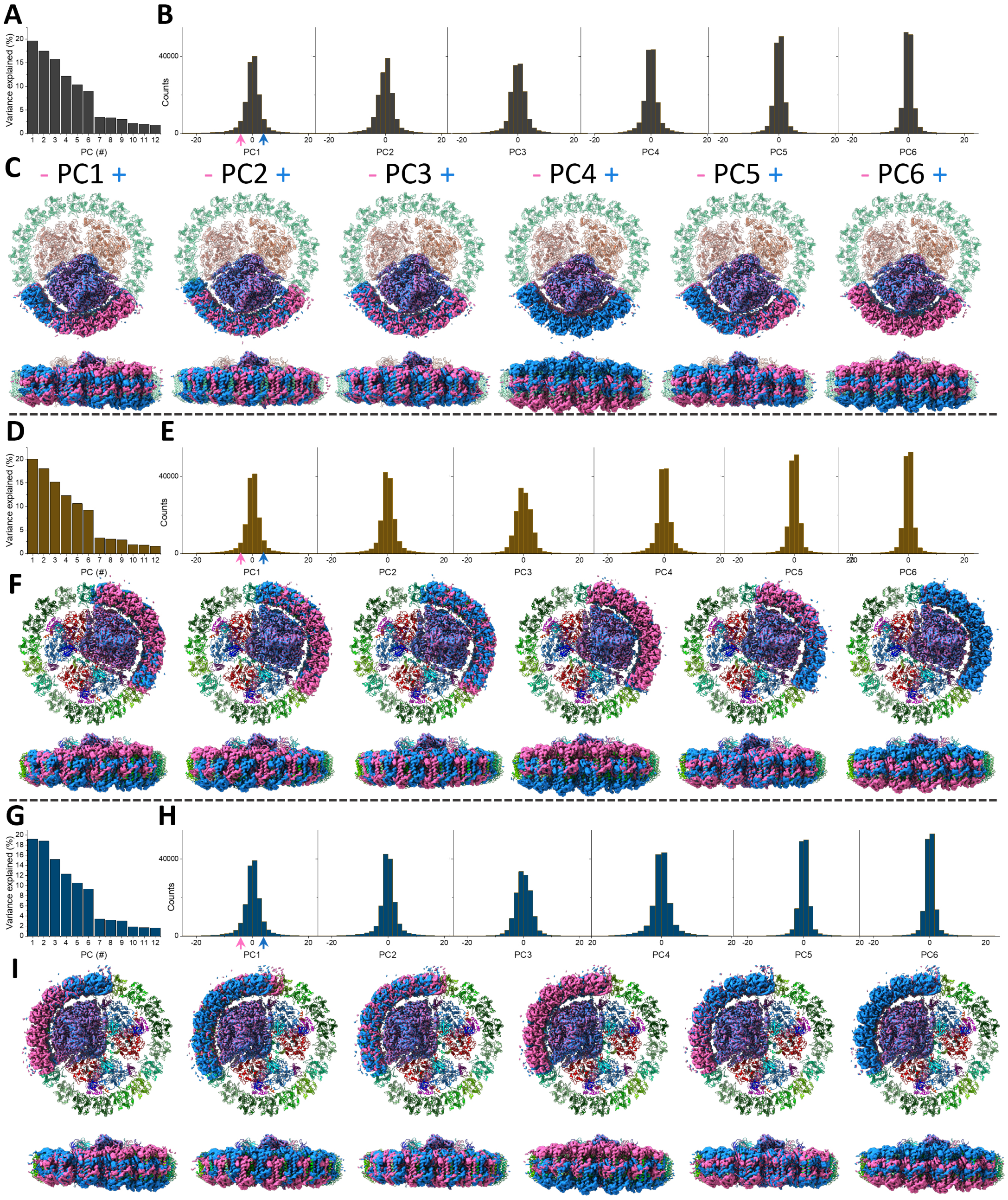

Supplementary Figure 5: Comparing principal components across the complete PSI-IsiA. (A, D, G) PC histograms of the explained variance. **(B, E, H)** particle distribution along each PC. The pink and blue arrows on the first histogram indicate the PC value of 9% and 91% of the particle population which was reconstructed as pink and blue maps. **(C, F, I)** Maps of PSI and IsiA$_6$ reconstructed at 9% and 91% PC values of each PC (viewed from the stromal side and the membrane side), shows that approximately similar shaped PC were found for each refinement (all done on the C3 expanded dataset). Due to hardware limitations refinement was carried out on a third of the complex at a time.

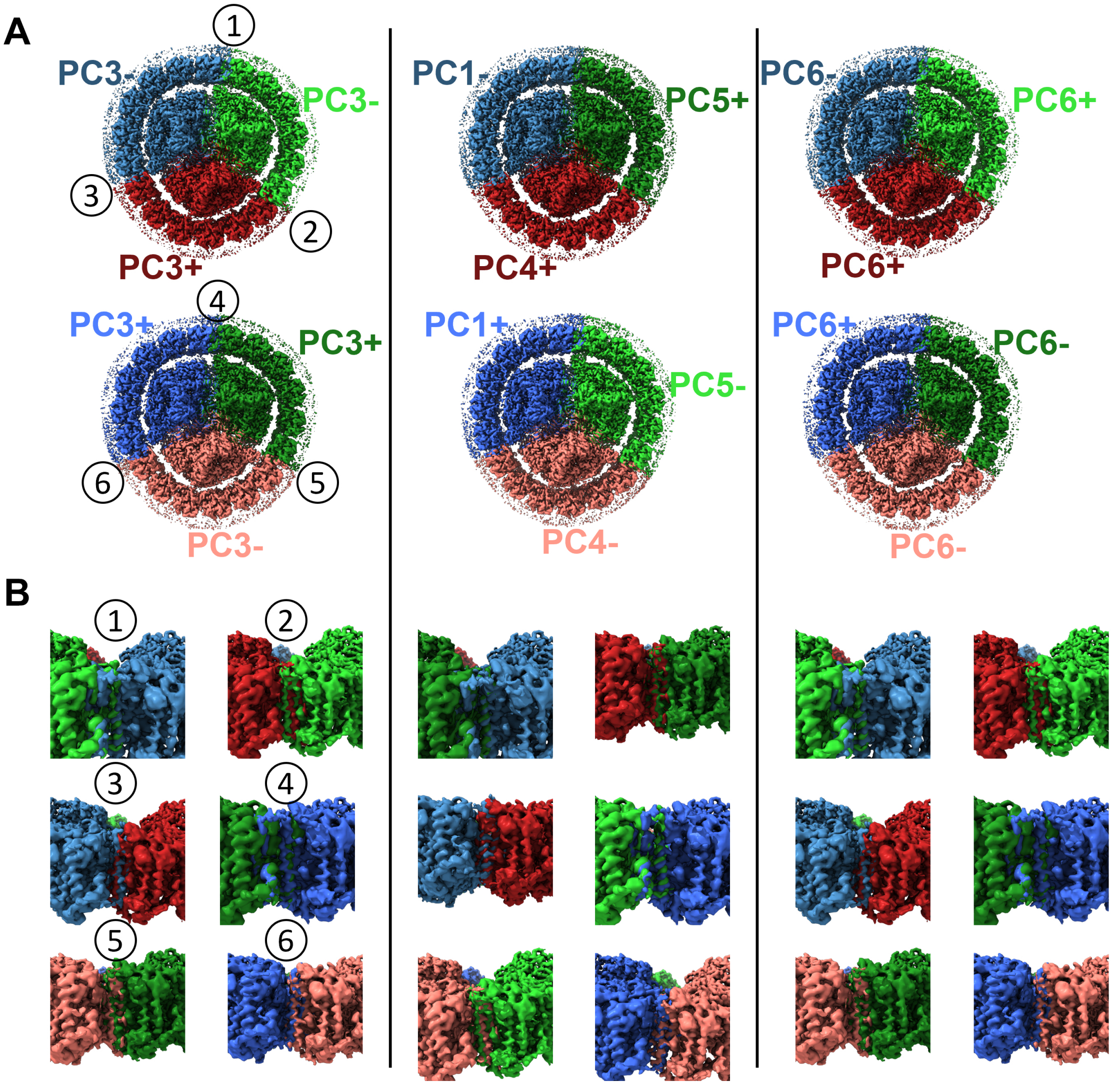

Supplementary Figure 6: Combining principal components into whole complexes. **(A)** PSI-IsiA thirds are colored differently to represent each independently analyzed third, for each third, a darker shade represent the negative side of PC axis and the lighter shade the positive side. The top row shows the initial state of PSI-IsiA. For example, the first PSI-IsiA reconstruction utilizes PC3 starting from the negative side on the blue and green thirds and PC3 from the positive side on the red third. The bottom image shows the PC combination in the final state with the blue and green thirds ending on the positive side and the red third ending on the negative side. Circled numbers represent the positions of the side views shown on the bottom part. **(B)** Side views on the overlap regions between the three independently analyzed regions of PSI-IsiA. Typically both masks overlap within a single trans membrane helix and this overlap was preserved for all aligned states along the different PC's. Vertical alignment between helices was not always perfectly maintained along the entire PCs, but in general did not exceed more then one helix turn.

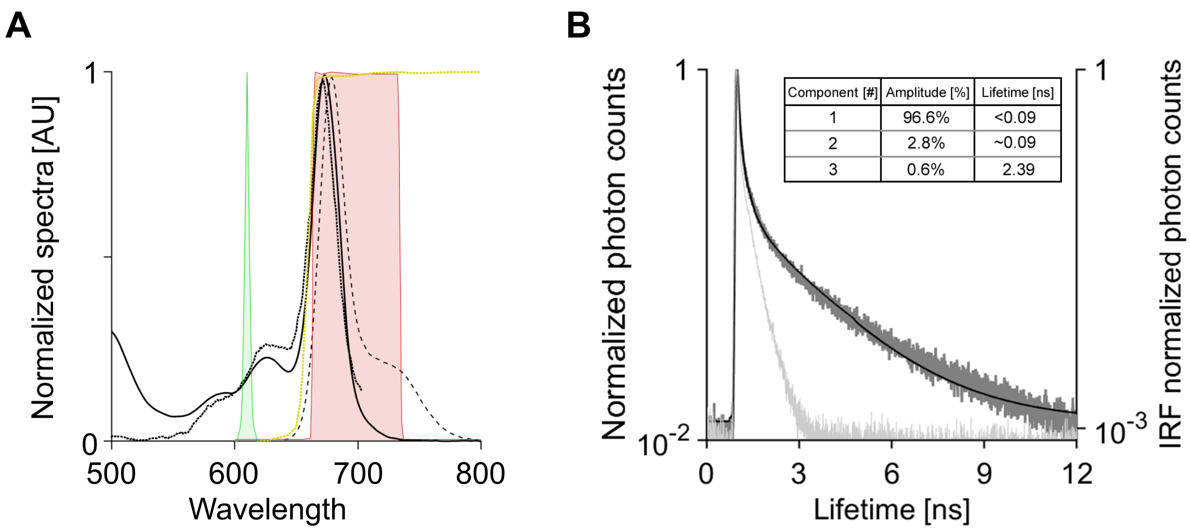

Supplementary Figure 7: Ensemble characterization of IsiA-PSI. (A) Linear absorption (solid black line), fluorescence excitation ($\lambda_{emission}$ = 720 nm, dotted line) and fluorescence emission ($\lambda_{excitation}$ = 610 nm, dashed line) of IsiA-PSI were normalized at 680 nm. dotted yellow line, λ = 607-613 nm green shade area and λ = 665-731 nm red shade area are the dichroic, excitation and emission filters, respectively, that were used for single molecule measurements. **(B)** Time-correlated Single Photon Counting (TSCPC) showing fluorescence decay of IsiA-PSI (dark gray) and its appropriate fit (black). Instrument response function (IRF; $\sim$90ps, light gray) is also shown. Table insert indicates the tri-exponential fit with the appropriate lifetimes and corresponding amplitudes. Y axes are in logarithmic scale.

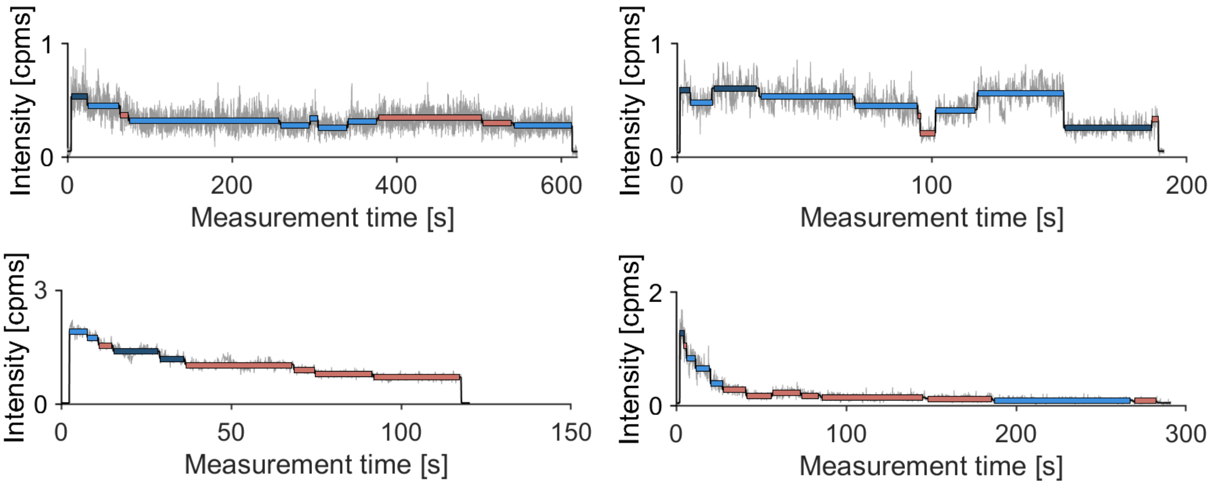

Supplementary Figure 8: Additional transient single particles intensity traces. Examples of single molecule intensity traces of transiently coupled complexes are shown. levels of mono-exponential fluorescence lifetime fast decay (pale red) are interleaved with bi-exponential fluorescence decay, having one component shorter than IRF and the other component grater than 1 ns (two different biexponential populations are indicated by light blue and dark blue).

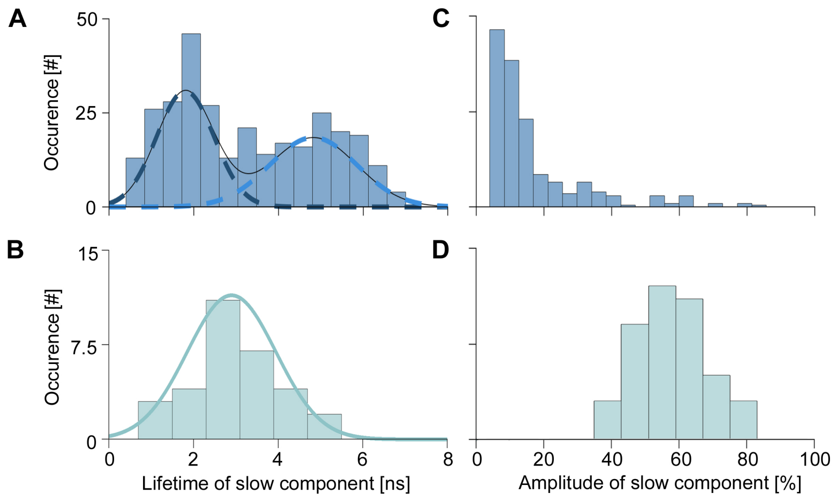

Supplementary Figure 9: Lifetime and amplitude distributions of slow component in biexponential states. Lifetime distribution of the slow component (of the bi-exponential fit) of either **(A)** 'transiently coupled' or **(B)** 'partially coupled' particles. 'Transiently coupled' particles showing two sub-populations (dark blue and light blue). with median lifetimes of 1.7 and 4.7 ns. Amplitude distribution of the slow component (of the bi-exponential fit) of either **(C)** 'transiently coupled' or **(D)** 'partially coupled' particles. 'Transiently coupled' particles showing much lower values compared to 'partially coupled'.

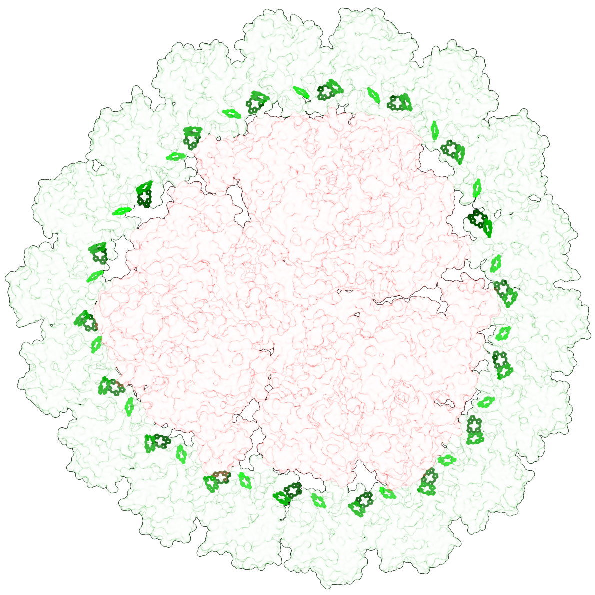

Supplementary Figure 10: IsiA Chls at the PSI-IsiA interface. Spatial positions of chl 8 (green), chl 17 (dark green) and chl 14 (light green) within the PSI-IsiA complex. Phytol chain of chlorophyll was omitted for clarity.

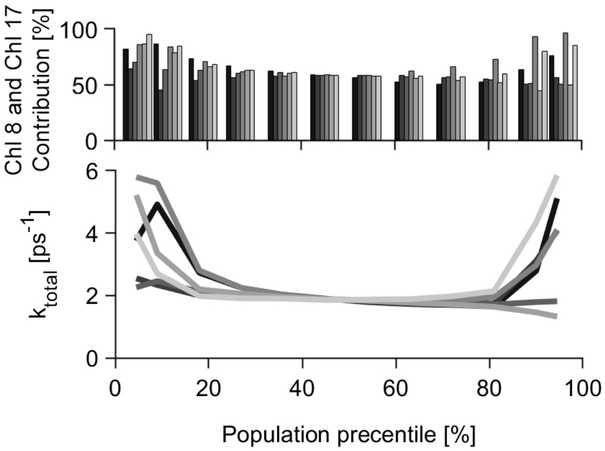

Supplementary Figure 11: Sum of rates at the PSI-IsiA interface (A) Bottom - For each principle component (PC1 to PC6, bright to dark gray), the sum of Förster rates from every IsiA and PSI chlorophyll pair under 25 Angström is plotted in PSI-IsiA models representing different population percentiles.

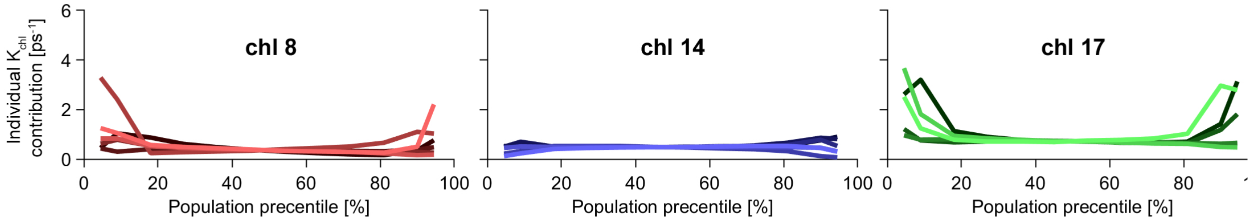

Supplementary Figure 12: Specific chlorophylls contribution to energy transfer rate (A) From left to right - chl 8 (reds), chl 14 (blues) and chl 17 (greens) contribution to overall energy transfer rate fron IsiA to PSI for every state in every PC (PC1 to PC6 - from dark to light).

II. Supplementary Tables

Supplementary Table 1: Cryo-EM data collection, refinement and validation statistics

.

Cryo-EM data collection, refinement and validation statistics

	(EMD-26601, PDB 7UMH)
**Data collection and processing**	
Magnification	47,600
Voltage (kV)	300
Electron exposure ($e^{-}/Å^2$)	59
Defocus range (μm)	0.5-3
Pixel size (Å)	0.525
Symmetry imposed	C1
Initial particle images (no.)	1,084,101
Final particle images* (no.)	47,913
Map resolution PSI, IsiA (Å)	2.6, 3.3
FSC threshold	0.143
Map resolution range (Å)	2.2 - 6
**Refinement**	
Initial model used (PDB code)	6NWA
Model resolution (Å)	2.6
FSC threshold	0.5
Model resolution range (Å)	2.2 - 60
Map sharpening *B* factor PSI, IsiA ($Å^2$)	-51, -69
Model composition	
Nonhydrogen atoms	140,516
Protein residues	12,747
Ligands	798
B factors ($Å^2$)	
Protein	66
Ligand	42
Water	26
R.m.s. deviations	
Bond lengths (Å)	0.005
Bond angles (°)	0.911
**Validation**	
MolProbity score	1.7
Clashscore	7.9
Poor rotamers (%)	0
Ramachandran plot	
Favored (%)	96.1
Allowed (%)	3.8
Disallowed (%)	0.1

* - before C3 expansion.

Supplementary Table 2: Fastest IsiA-PSI FRET pair in selected percentiles across the top 6 PCs

.

	PC1		PC2		PC3		PC4		PC5		PC6	
	k_{FRET} [ps^{-1}] IsiA$_a$-PsaK Chl_{17} - Chl_{4003}	k_{FRET} [ps^{-1}] IsiA$_d$-PsaJ Chl_{17} - Chl_{1303}	k_{FRET} [ps^{-1}] IsiA$_a$-PsaK Chl_{17} - Chl_{4003}	k_{FRET} [ps^{-1}] IsiA$_d$-PsaJ Chl_{17} - Chl_{1303}	k_{FRET} [ps^{-1}] IsiA$_a$-PsaK Chl_{17} - Chl_{4003}	k_{FRET} [ps^{-1}] IsiA$_d$-PsaJ Chl_{17} - Chl_{1303}	k_{FRET} [ps^{-1}] IsiA$_a$-PsaK Chl_{17} - Chl_{4003}	k_{FRET} [ps^{-1}] IsiA$_d$-PsaJ **Chl_8** - Chl_{1303}	k_{FRET} [ps^{-1}] IsiA$_a$-PsaK Chl_{17} - Chl_{4003}	k_{FRET} [ps^{-1}] IsiA$_d$-PsaJ Chl_{17} - Chl_{1303}	k_{FRET} [ps^{-1}] IsiA$_a$-PsaK Chl_{17} - Chl_{4003}	k_{FRET} [ps^{-1}] IsiA$_d$-PsaJ **Chl_8** - Chl_{1303}
**5%**	2.14	0.02	0.01	0.79	0.72	0.03	0.01	2.43	1.72	1.23	2.3	0.01
**50%**	0.29	0.02	0.29	0.02	0.29	0.02	0.29	0.01	0.29	0.02	0.29	0.02
**95%**	0.01	1.03	0.92	0.32	0.13	0.08	2.72	0.01	0.05	0.01	0.03	2.05
