# Supplementary material for: Energetic robustness to large scale structural fluctuations in a photosynthetic supercomplex
Source: Nat Commun. 2023 Aug 2;14:4650. doi: 10.1038/s41467-023-40146-8 (PMC10397321; doi:10.1038/s41467-023-40146-8)
Supplement: Supplementary file 3 — Description of Additional Supplementary Files [file 41467_2023_40146_MOESM3_ESM.pdf]

Description of Additional Supplementary Files

File name: Supplementary Movie 1

Description: Whole complex structural transformation using PC3, 3 and 3 across all thirds.

File name: Supplementary Movie 2

Description: Whole complex structural transformation using PC4, 5 and 1 across all thirds.

File name: Supplementary Movie 3

Description: Whole complex structural transformation using PC6, 6 and 6 across all thirds.
