# Supplementary material for: Energetic robustness to large scale structural fluctuations in a photosynthetic supercomplex
Source: Nat Commun. 2023 Aug 2;14:4650. doi: 10.1038/s41467-023-40146-8 (PMC10397321; doi:10.1038/s41467-023-40146-8)
Supplement: Supplementary file 7 — Reporting Summary [file 41467_2023_40146_MOESM7_ESM.pdf]

nature portfolio

Corresponding author(s): Yuval Mazor

Last updated by author(s): Jun 22, 2023

Reporting Summary

Nature Portfolio wishes to improve the reproducibility of the work that we publish. This form provides structure for consistency and transparency in reporting. For further information on Nature Portfolio policies, see our Editorial Policies and the Editorial Policy Checklist.

Statistics

For all statistical analyses, confirm that the following items are present in the figure legend, table legend, main text, or Methods section.

n/a	Confirmed	
☐	☒	The exact sample size (*n*) for each experimental group/condition, given as a discrete number and unit of measurement
☐	☒	A statement on whether measurements were taken from distinct samples or whether the same sample was measured repeatedly
☐	☒	The statistical test(s) used AND whether they are one- or two-sided<br>*Only common tests should be described solely by name; describe more complex techniques in the Methods section.*
☒	☐	A description of all covariates tested
☒	☐	A description of any assumptions or corrections, such as tests of normality and adjustment for multiple comparisons
☐	☒	A full description of the statistical parameters including central tendency (e.g. means) or other basic estimates (e.g. regression coefficient) AND variation (e.g. standard deviation) or associated estimates of uncertainty (e.g. confidence intervals)
☐	☒	For null hypothesis testing, the test statistic (e.g. *F*, *t*, *r*) with confidence intervals, effect sizes, degrees of freedom and *P* value noted<br>*Give P values as exact values whenever suitable.*
☒	☐	For Bayesian analysis, information on the choice of priors and Markov chain Monte Carlo settings
☒	☐	For hierarchical and complex designs, identification of the appropriate level for tests and full reporting of outcomes
☒	☐	Estimates of effect sizes (e.g. Cohen's *d*, Pearson's *r*), indicating how they were calculated

Our web collection on statistics for biologists contains articles on many of the points above.

Software and code

Policy information about availability of computer code

Data collection	SerialEM3.8 – automated image acquisition.
Data analysis	MotionCor2 – correction of beam induced motion and radiation exposure.<br>CTFFIND4 – estimation of ctf parameters for micrographs and particles.<br>Relion4 - 2D and 3D classification, ab-initio alignment, 3D refinement of particle alignment, mask calculation, map sharpening.<br>Topaz 0.24 - particle picking.<br>ResMap1.14 – local resolution estimate.<br>Phenix1.20-4438 – real space restrained refinement and validation.<br>Coot0.96 – manual model building, real space refinement and validation.<br>Pymol2.7 – image preparation and distance calculations.<br>uscfChimeraX1.4 - image preparation.<br>Origin2017 - numerical data presentation and analysis.<br>Matlab R2019b - spectroscopic analyses and plots generation.<br>Rstudio - theoretical FRET calculations.

For manuscripts utilizing custom algorithms or software that are central to the research but not yet described in published literature, software must be made available to editors and reviewers. We strongly encourage code deposition in a community repository (e.g. GitHub). See the Nature Portfolio guidelines for submitting code & software for further information.

Data

Policy information about availability of data

All manuscripts must include a data availability statement. This statement should provide the following information, where applicable:

- Accession codes, unique identifiers, or web links for publicly available datasets
- A description of any restrictions on data availability
- For clinical datasets or third party data, please ensure that the statement adheres to our policy

The cryo-EM density maps were deposited in the Electron Microscopy Data Bank (EMDB, www.ebi.ac.uk/pdbe/emdb/) under the following accession codes: EMD-26601. The atomic coordinates have been deposited in the Protein Data Bank (PDB, www.rcsb.org) under the following accession codes: 7UMH. MATLAB and R code used to calculate Förster rates and perform additional data analysis are available from the authors upon request.

Human research participants

Policy information about studies involving human research participants and Sex and Gender in Research.

Reporting on sex and gender	N/A
Population characteristics	N/A
Recruitment	N/A
Ethics oversight	N/A

Note that full information on the approval of the study protocol must also be provided in the manuscript.

Field-specific reporting

Please select the one below that is the best fit for your research. If you are not sure, read the appropriate sections before making your selection.

☒ Life sciences ☐ Behavioural & social sciences ☐ Ecological, evolutionary & environmental sciences

For a reference copy of the document with all sections, see nature.com/documents/nr-reporting-summary-flat.pdf

Life sciences study design

All studies must disclose on these points even when the disclosure is negative.

Sample size	Approximately 44,000 individual views of PSI-IsiA were analyzed to measure population distribution.
Data exclusions	The initial set of particles picked during cryoEM analysis consists of app. 1,000,000 particles. This set was reduced to ~44,000 particles using classification techniques as common in the field. The steps taken during data analysis are described in Supplementary fig. 1
Replication	The cryoEM analysis in this study was not replicated.
Randomization	During refinement particles are randomly split into two groups and refined independently to obtain an unbiased resolution estimates.
Blinding	No blinding was performed as part of data analaysis

Reporting for specific materials, systems and methods

We require information from authors about some types of materials, experimental systems and methods used in many studies. Here, indicate whether each material, system or method listed is relevant to your study. If you are not sure if a list item applies to your research, read the appropriate section before selecting a response.

Materials & experimental systems

n/a	Involved in the study
☒	☐ Antibodies
☒	☐ Eukaryotic cell lines
☒	☐ Palaeontology and archaeology
☒	☐ Animals and other organisms
☒	☐ Clinical data
☒	☐ Dual use research of concern

Methods

n/a	Involved in the study
☒	☐ ChIP-seq
☒	☐ Flow cytometry
☒	☐ MRI-based neuroimaging
